# Medical management patterns in a US commercial claims database following a nontraumatic fracture in postmenopausal women

**DOI:** 10.1007/s11657-022-01135-4

**Published:** 2022-07-14

**Authors:** Xin Wang, Xiaoqing Xu, Mary Oates, Timothy Hill, Rolin L. Wade

**Affiliations:** 1 IQVIA, Plymouth Meeting, PA USA; 2 Amgen Inc., Thousand Oaks, CA USA

**Keywords:** Osteoporosis, Fracture, Diagnosis, Treatment, Hospitalization, Surgery

## Abstract

**Summary:**

Among women ≥ 50 years with fracture, 76% had not received osteoporosis diagnosis or treatment at 6 months and only 14% underwent a DXA scan. Nearly half of all and 90% of hip fracture patients required surgery. Fractures cause substantial clinical burden and are not linked to osteoporosis diagnosis or treatment.

**Purpose:**

Osteoporosis (OP) and OP-related fractures are a major public health concern, associated with significant economic burden. This study describes management patterns following a nontraumatic fracture for commercially insured patients.

**Methods:**

This retrospective cohort study identified women aged ≥ 50 years having their first nontraumatic index fracture (IF) between January 1, 2015 and June 30, 2019, from IQVIA’s PharMetrics® Plus claims database. Medical management patterns at month 6 and medication use patterns at months 6, 12, and 24 following the IF were described.

**Results:**

Among 48,939 women (mean (*SD*) age: 62.7 (9.5) years), the most common fracture types were vertebral (30.6%), radius/ulna (24.9%), and hip (HF; 12.1%). By month 6, 76% of patients had not received an OP diagnosis or treatment, 13.6% underwent a DXA scan, and 11.2% received any OP treatment. Surgery was required in 43.1% of all patients and 90.0% of HF patients on or within 6 months of the fracture date. Among HF patients, 41.4% were admitted to a skilled nursing facility, 96.7% were hospitalized an average of 5.5 days, and 38.1% required durable medical equipment use. The 30-day all-cause readmission rate was 14.3% among those hospitalized for the IF. Overall, 7.4%, 9.9%, and 13.2% had a subsequent fracture at months 6, 12, and 24, respectively.

**Conclusion:**

Our findings provide an overview of post-fracture management patterns using real-world data. OP was remarkably underdiagnosed and undertreated following the initial fracture. Nontraumatic fracture, particularly HF, resulted in substantial ongoing clinical burden.

**Supplementary Information:**

The online version contains supplementary material available at 10.1007/s11657-022-01135-4.

## Background

Osteoporosis (OP) and OP-related fractures are a major public health problem in the USA, which reduce quality of life (QoL), increase psycho-social impairment, and increase financial expenditures [1, 2]. Fracture is associated with an increased rate of hospital admissions, utilization of nursing home and rehabilitation facilities, and an annual societal cost including both direct medical costs and indirect costs of approximately 57 billion USD in 2018, which is projected to increase to over $95 billion in 2040 with a growing aging US population [3–5].

In women, the lifetime risk of sustaining a nontraumatic fracture has been estimated around 40 to 50% [4, 6]. Risk factors for OP-related fractures include history of falls and fracture, smoking and alcohol use, some specific diseases, low calcium intake, and the use of certain classes of medications [4]. Patients with a history of fracture are 80% more likely to experience a subsequent fracture compared to those without prior fracture history, which increases the medical costs by 2–6 times [7–9]. Early diagnosis of OP and initiation of pharmacotherapy play a significant role in reducing fracture rate and relevant costs [10, 11]. The American Association of Clinical Endocrinologists (AACE) guidelines recommend pharmacotherapy for the patients with OP considered “high” or “very high” risk of fracture [12]. While those who experience a nontraumatic fracture are considered to be at very high risk of fracture, most patients presenting with incident nontraumatic fractures are neither assessed nor treated for osteoporosis to reduce their risk of subsequent fractures, despite the availability of effective treatments [13]. Recent reports indicate that only about 20% of patients with a fracture receive treatment to reduce the risk of subsequent fractures [14, 15]. Effective management of these fractures requires appropriate management of the underlying osteoporosis along with acute treatment for fractures [13].

Management patterns of nontraumatic fracture have been explored to some extent in the real-world setting, mostly among patients enrolled in public health plans [16–18]. One analysis in fee-for-service Medicare patients reported post-acute care utilization measures such as home health, outpatient visits, and rehabilitation, but outpatient pharmacy utilization was not assessed [17]. Another inpatient analysis of Medicare patients with hip fracture reported the type of inpatient treatment (e.g. fixation, arthroplasty, etc.) as well as inpatient rehabilitation measures [18]. However, this analysis was not extended to report medical or pharmacy utilization in the outpatient setting among the 73.2% of patients who were ultimately discharged.

Liu et al. previously assessed the clinical and economic burden of osteoporotic fracture among elderly female Medicare beneficiaries and found that less than 30% of the fracture cohort had osteoporosis medication use in the first year of follow-up [19]. These prior studies focused on Medicare beneficiaries; data are lacking for the medical management patterns following a nontraumatic fracture for commercially insured patients. This study addresses this knowledge gap and helps develop a holistic understanding of nontraumatic fracture management by analyzing real-world management patterns.

## Methods

### Study design and databases

This was a retrospective cohort study using IQVIA PharMetrics® Plus health plan claims database. The aggregated IQVIA PharMetrics Plus database is a nationally representative commercial claims database which comprises adjudicated medical and pharmacy claims of patients in the USA and is sourced directly from the commercial payers [20]. In addition to those with other commercial health plans (HMO, PPO etc.), the database also includes patients eligible for Medicare Advantage plans and does not include those eligible for Medicare fee-for-service, with or without Medigap Part B coverage. The overall study period was from January 1, 2014 to December 31, 2019. The index period was from January 1, 2015 to June 30, 2019, to allow for 1-year pre-index and a minimum of 6-month post-index period, with the index date defined as the first occurrence of hip, vertebral, or non-hip non-vertebral (NHNV) fracture in the inpatient or outpatient setting during the index period. NHNV fracture included fracture of ankle, clavicle, femur, humerus, pelvis, radius/ulna, and tibia/fibula. Patients’ demographic and baseline clinical characteristics were assessed during the 1-year pre-index period. The medical management patterns were described at month 6 and treatment patterns were reported at months 6, 12, and 24 following the index nontraumatic fracture.

### Inclusion and exclusion criteria

**Fig. 1:**
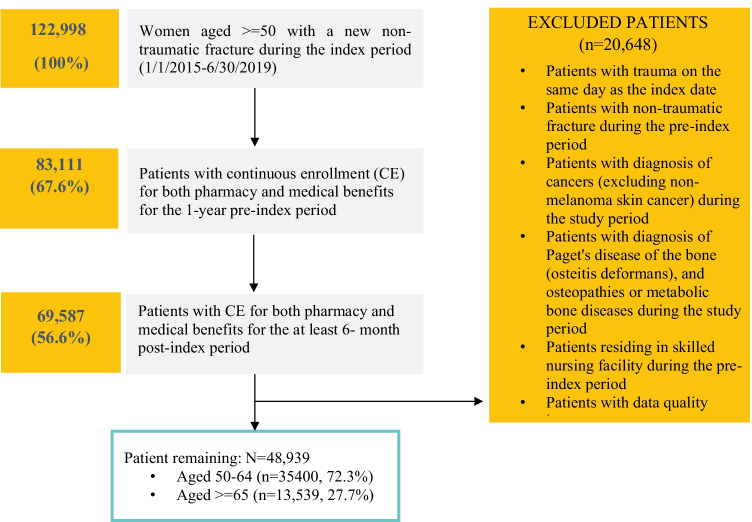
Patient attrition

Patients eligible for inclusion were women aged 50 years and older with a hip, vertebral, or NHNV fracture in the inpatient or outpatient settings between January 1, 2015 and June 30, 2019 (Fig. 1). Patients were required to be continuously enrolled with both medical and outpatient pharmacy benefits during the 1-year period prior to the index fracture date and for at least 6-month post-index. Patients with trauma diagnosis on the same day as index fracture, or with pre-index nontraumatic fracture or with a diagnosis of Paget’s disease of bone, osteitis deformans, known primary bone diseases other than postmenopausal OP, or metabolic bone diseases, or with a pre-index diagnosis of cancer (excluding non-melanoma skin cancer) during the study period were excluded from the study.

The fracture identification algorithm identified nontraumatic fractures and employed a combination of ICD-9-CM or ICD-10-CM diagnosis codes and Current Procedural Terminology (CPT) codes and required at least one claim with a relevant ICD-9-CM or ICD-10-CM diagnosis code in either the inpatient or outpatient setting. For outpatient claims, additionally, a corresponding procedure code for fracture treatment at the same anatomic site was also required; with the exception being vertebral fracture, where a corresponding diagnosis code with CPT codes for outpatient physician evaluation and management was sufficient. If there were two or more fractures on the same index date, index fracture was assigned according to the following hierarchy: hip, femur, pelvis, vertebral, humerus, radius/ulna, tibia/fibula, ankle, and clavicle [21]. Subsequent fractures occurring after the index fracture were considered a new fracture if they occurred at a new anatomical site or at the same site as a previous fracture and occurring more than 90-days from the original fracture.

### Baseline patient characteristics

Patients’ demographics including age, geographic region, payer type, and clinical characteristics (pre-index claim(s) for DXA scan, pre-index history of fall, pre-index OP diagnosis, pre-index orthopedic surgery, pre-index use of OP medications, pre-index history of smoking, corticosteroid use, Charlson comorbidity index (CCI), and other comorbidities of interest (e.g., osteoarthritis, rheumatoid arthritis, Alzheimer/dementia, depression, ischemic stroke, and other cardiovascular diseases including myocardial infarction) were reported during the 1-year pre-index period.

### Study outcomes

Medical management patterns including index fracture hospitalization, post index OP diagnosis, DXA scan, orthopedic surgery, rehabilitation, and all-cause readmission within 30 days post discharge of the hospitalization for index fracture were described at 6 months following the index nontraumatic fracture. Treatment patterns including use of OP medications, discontinuation, and re-initiation of the first OP agent (anabolic agents (teriparatide, abaloparatide, romosozumab) or antiresorptive agents (bisphosphonates, denosumab, selective estrogen receptor modulators SERMs, calcitonin)) following the index nontraumatic fracture, and total months on OP treatment (not including treatment gaps) were described at 6, 12, and 24 months following the index nontraumatic fracture. In addition, occurrence of new subsequent fractures was also evaluated at 6, 12, and 24 months following the index nontraumatic fracture. Discontinuation was defined as an observation of a gap of > 60 days between the end of days of supply and the next fill of the first OP drug class that was initiated following the index fracture event. The last day of medication supply was defined as the discontinuation date. Re-initiation was defined as starting the same drug class after discontinuation (discontinuation and re-initiation were defined at drug class level.)

This retrospective study used only existing de-identified aggregate claims data, therefore informed consent, ethics committee approval, or institutional review board approval were not required. The study complied with all applicable laws regarding patient privacy, using HIPAA-compliant de-identified retrospective data sources.

### Statistical analysis

Analyses were conducted using SAS version 9.3 (SAS Institute, Cary, NC, USA). The study was descriptive in nature and formal statistical tests were not conducted. Mean and standard deviation (SD) were generated as measures of central tendency and variance for continuous variables. Frequencies and percentages were calculated for categorical variables.

## Results

**Fig. 2:**
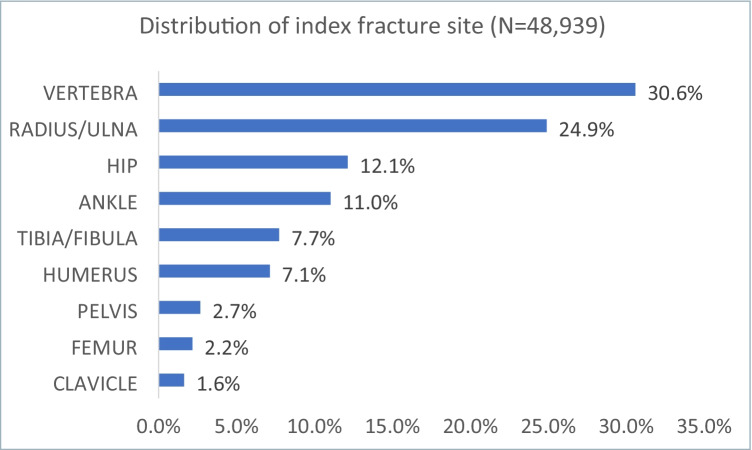
Distribution of index fracture

Of the 48,939 women (mean (*SD*) age: 62.7 (9.5) years) included in the analysis, 13,539 (27.7%) were ≥ 65 years old (Table 1). The most common index fracture type was vertebral (30.6%), followed by radius/ulna (24.9%) and hip (12.1%) (Fig. 2). The inpatient setting was reported for 34.8% of the index fractures. Among all women with incident nontraumatic fractures, 10.8% had OP diagnosis prior to index fracture; 9.7% had DXA scan and 7.3% received OP treatment in the 12-month pre-index period (Table 2).

**Table 1:** Patients demographic characteristics

Baseline demographic characteristics	Overall cohort (*N* = 48,939)	With post index OP claim by 6 month (*N* = 5,473)	Without post index OP claim by 6 month (*N* = 43,466)
Age group, *N* (%)			
50–59	21,341 (43.6)	1451 (26.5)	19,890 (45.8)
60–64	14,059 (28.7)	1687 (30.8)	12,372 (28.5)
65–70	4901 (10.0)	755 (13.8)	4146 (9.5)
71–79	3440 (7.0)	685 (12.5)	2755 (6.3)
≥ 80	5198 (10.6)	895 (16.4)	4303 (9.9)
Age, mean (*SD*)	62.7 (9.5)	66.3 (9.8)	62.3 (9.4)
Geographic region			
Northeast	10,762 (22.0)	1081 (19.8)	9681 (22.3)
Midwest	12,691 (25.9)	1323 (24.2)	11,368 (26.2)
South	17,115 (35.0)	1750 (32.0)	15,365 (35.3)
West	8371 (17.1)	1319 (24.1)	7052 (16.2)
Payer type, *N* (%)			
Commercial	42,677 (87.2)	4452 (81.3)	38,225 (87.9)
Medicare risk	2955 (6.0)	637 (11.6)	2318 (5.3)
Medicaid	1993 (4.1)	210 (3.8)	1783 (4.1)
Unknown	1314 (2.7)	174 (3.2)	1140 (2.6)
Health plan type, *N* (%)			
HMO	8147 (16.6)	1129 (20.6)	7018 (16.1)
PPO	36,765 (75.1)	3933 (71.9)	32,832 (75.5)
POS	2395 (4.9)	216 (3.9)	2179 (5.0)
Consumer-directed	58 (0.1)	6 (0.1)	52 (0.1)
Indemnity	1202 (2.5)	156 (2.9)	1046 (2.4)
All other	372 (0.8)	33 (0.6)	339 (0.8)

**Table 2:** Clinical characteristics

Baseline clinical characteristics	Overall cohort (*N* = 48,939)	With post index OP claim by 6 month (*N* = 5,473)	Without post index OP claim by 6 month (*N* = 43,466)
CCI, mean (*SD*)	0.8 (1.3)	1.0 (1.4)	0.8 (1.3)
CCI category, *N* (%)			
0	28,716 (58.7)	2738 (50.0)	25,978 (59.8)
1	10,637 (21.7)	1387 (25.3)	9250 (21.3)
2 +	9586 (19.6)	1348 (24.6)	8238 (19.0)
Diagnosis of OP, *N* (%)	5292 (10.8)	2117 (38.7)	3175 (7.3)
OP diagnosis or treatment*, N* (%)	6583 (13.5)	3024 (55.3)	3559 (8.2)
DXA scan, *N* (%)	4736 (9.7)	1290 (23.6)	3446 (7.9)
Time inteval (days) between last DXA and index, mean (*SD*), *N*	172.6 (106.8), 4736	161.2 (107.0), 1290	176.8 (106.4), 3446
≤ 30 days, *N* (%)*	534 (11.3)	192 (14.9)	342 (9.9)
> 30–60 days	446 (9.4)	116 (9.0)	330 (9.6)
> 60–180 days	1507 (31.8)	428 (33.2)	1079 (31.3)
> 180–360 days	2249 (47.5)	554 (42.9)	1695 (49.2)
Pre-index orthopedic surgery, *N* (%)	895 (1.8)	97 (1.8)	798 (1.8)
Time inteval (days) between last orthopedic surgery and index, mean (*SD*), *N*	126.5 (115.3), 895	139.6 (139.6), 97	124.9 (124.9), 798
≤ 30 days, *N*** (%)**^**†**^	291 (32.5)	28 (28.9)	263 (33.0)
> 30–60 days	82 (9.2)	4 (4.1)	78 (9.8)
> 60–180 days	229 (25.6)	28 (28.9)	201 (25.2)
> 180–360 days	293 (32.7)	37 (38.1)	256 (32.1)
Pre-index OP med use, *N* (%)	3562 (7.3)	2454 (44.8)	1108 (2.5)
Anabolic agents	101 (0.2)	68 (1.2)	33 (0.1)
Anti-resorptive agents	3481 (7.1)	2398 (43.8)	1083 (2.5)
Smoking, *N* (%)	3999 (8.2)	585 (10.7)	3414 (7.9)
Osteoarthritis, *N* (%)	9723 (19.9)	1385 (25.3)	8338 (19.2)
Rheumatoid arthritis, *N* (%)	1369 (2.8)	321 (5.9)	1048 (2.4)
Alzheimer/dementia, *N* (%)	466 (1.0)	66 (1.2)	400 (0.9)
Parkinson, *N* (%)	327 (0.7)	66 (1.2)	261 (0.6)
Multiple sclerosis, *N* (%)	430 (0.9)	62 (1.1)	368 (0.8)
Other central nervous system disease, *N* (%)	1565 (3.2)	212 (3.9)	1353 (3.1)
Vertigo, *N* (%)	3463 (7.1)	465 (8.5)	2998 (6.9)
Depression, *N* (%)	8819 (18.0)	1001 (18.3)	7818 (18.0)
Anxiety, *N* (%)	9093 (18.6)	969 (17.7)	8124 (18.7)
Other psychoses, *N* (%)	2013 (4.1)	270 (4.9)	1743 (4.0)
Blood pressure abnormalities, *N* (%)	22,445 (45.9)	2754 (50.3)	19,691 (45.3)
Cardiovascular disease, *N* (%)	14,496 (29.6)	1896 (34.6)	12,600 (29.0)
Ischemic stroke (IS), *N* (%)	822 (1.7)	101 (1.8)	721 (1.7)
Other cerebrovascular events, *N* (%)	1794 (3.7)	260 (4.8)	1534 (3.5)
Alcohol/drug abuse, *N* (%)	4047 (8.3)	439 (8.0)	3608 (8.3)
Pre-index history of falls, *N* (%)	1163 (2.4)	142 (2.6)	1021 (2.3)
Thyroid and parathyroid disorders, *N* (%)	849 (1.7)	139 (2.5)	710 (1.6)
Thyroid-related medications, *N* (%)	9255 (18.9)	1276 (23.5)	7979 (18.4)
DME use, *N* (%)	1596 (3.3)	184 (3.4)	1412 (3.2)
Other pre-index medicaiton use, *N* (%)			
Narcotics	15,919 (32.5)	2107 (38.5)	13,812 (31.8)
Antidepressants	14,390 (29.4)	1734 (31.7)	12,656 (29.1)
Muscle relaxants	6877 (14.1)	1001 (18.3)	5876 (13.5)
Tranquilizers	1251 (2.6)	141 (2.6)	1110 (2.6)
Sedatives	2968 (6.1)	372 (6.8)	2596 (6.0)
Benzodiazepines	11,826 (24.2)	1433 (26.2)	10,393 (23.9)
Anti-Parkinson agents	1242 (2.5)	175 (3.2)	1067 (2.5)
Anti-hypertensive	21,955 (44.9)	2812 (51.4)	19,143 (44.0)
6-month pre-index corticosteroid use, *N* (%)	10,715 (21.9)	1541 (28.2)	9174 (21.1)

^*^% represents n/(number of patients with pre-index DXA in row above). ^†^% represents n/(number of patients with pre-index orthopedic surgery in row above)

### Management patterns

By month 6 post index nontraumatic fracture, 76% of the patients had not received an OP diagnosis or any treatment for OP, 13.6% underwent a DXA scan, 12.4% of the patients were admitted to a skilled nursing facility (SNF), and 17.1% required DME use within 6 month from the index fracture event. Among the patients who received OP treatment prior to index fracture, the post index DXA scan rate was 15.9% and 40.0% had orthopedic surgery within 6 months from the index fracture. Among the patients with a hip fracture, 90.0% had orthopedic surgery within 6 months of the index fracture, 41.4% were admitted to a SNF, 96.7% were hospitalized for an average of 5.5 days, and 38.1% required durable medical equipment (DME) use within 6 months from the index fracture. The all-cause hospital readmission rate following discharge from the index hospitalization was 14.3% for overall patients and 15.1% for the patients with index hip fracture (Table 3).

**Table 3:** Post fracture management pattern by 6 months following index nontraumatic fracture among the overall patients and subgroups of interest

Post index measures by 6 months	Overall cohort (*N* = 48,939)	With index hip fracture (*N* = 5,935)	With index vetebral fracture (*N* = 14,970)	With index NHNV fracture (*N* = 28,034)	With pre-index OP diagnosis (*N* = 5,292)	With pre-index OP med (*N* = 3,562)	With pre-index antiresorptive agents (*N* = 3,474)	With pre-index anabolic agents (*N* = 88)
Hospitalization for index nontraumatic fracture, %	34.8%	96.7%	21.3%	28.9%	37.1%	36.6%	36.8%	28.4%
LOS for index fracture hospitalizaiton, mean days (SD)	5.5 (4.1)	5.5 (3.4)	6.0 (4.7)	5.2 (4.2)	5.6 (3.9)	5.7 (3.9)	5.7 (3.9)	6.9 (5.8)
OP diagnosis, %	20.6%	37.0%	30.0%	12.1%	64.7%	63.5%	63.1%	78.4%
OP diagnosis or treatment, %	23.7%	39.8%	34.9%	14.3%	71.7%	85.3%	85.3%	84.1%
DXA scan, %	13.6%	13.6%	18.8%	10.9%	14.1%	15.7%	15.7%	15.9%
Time (days) to first DXA scan during the post-index period, mean (SD)	75.3 (50.8)	86.9 (45.5)	57.4 (49.4)	88.8 (48.4)	81.3 (53.2)	83.4 (52.4)	83.6 (52.5)	75.2 (48.2)
Post-index orthopedic surgery, %	43.1%	90.0%	17.0%	47.2%	38.6%	39.8%	40.1%	26.1%
Rehabilitation, %								
Physical or occupational therapy or home health care	56.4%	66.3%	39.8%	63.2%	54.6%	55.9%	56.2%	46.6%
SNF	12.4%	41.4%	8.2%	8.5%	17.9%	17.3%	17.5%	12.5%
Time (days) from index fracture to discharge to SNF, mean (SD)	14.2 (25.2)	9.4 (15.7)	24.8 (37.6)	13.6 (23.6)	14.8 (26.7)	14.2 (24.5)	14.2 (24.6)	11.9 (17.8)
All-cause readmission within 30 day following the discharge of index fracture hospitalization, %	14.3%	15.6%	13.0%	13.8%	16.8%	16.2%	16.3%	12.0%
DME use, %	17.1%	38.1%	9.4%	16.8%	16.6%	17.2%	17.2%	13.6%
Walkers	11.9%	32.9%	7.0%	10.1%	12.3%	12.3%	12.4%	9.1%
Wheelchairs	3.5%	6.9%	2.4%	3.4%	3.5%	4.0%	3.9%	4.5%
Other	3.7%	1.7%	0.8%	5.6%	2.1%	2.3%	2.4%	0.0%

*DME*, durable medical equipment; *DXA*, dual-energy X-ray absorptiometry; *HF*, hip fracture; *LOS*, length of stay; *NHNV*, non-hip-non-vertebral; *SNF*, skilled nursing facility; *VF*, vertebral fracture

### Treatment patterns

Overall, 11.2%, 14.3%, and 17.6% of the fracture patients received OP medications within 6, 12, and 24 months post index fracture regardless of their pre-index OP medication use status. Post-index OP medication utilization rate was 68.9–80.8% within 6–24 months following index fracture among the subgroup of patients with pre-index OP medication use. Among patients who were treated post fracture, only 6.3% (6.8%) received anabolic agents, and 93.7% (93.2%) received antiresorptive agents within 6 (12) months following index fracture. In the OP treatment-naïve group, which initiated OP medication within 6 months after fracture, the mean time to OP treatment initiation was 69 days and over 50% of these treatment-naïve patients initiated OP treatment more than 60 days after fracture (Table 4).

**Table 4:** Treatment pattern and occurrence of subsequent new fracture by 6, 12, and 24 months following index nontraumatic fracture among overall patients and subgroups of interest

	Overall cohort			Subgroup of patients with pre-index OP diagnosis			Subgroup of patients with pre-index OP medication			Subgroups of patients with pre-index antiresorptive agents			Subgroups of patients with pre-index anabolic agents		
Post index treatment measures	By 6 mon (*N* = 48,939)	By 12 mon (*N* = 37,255)	By 24 mon (*N* = 20,207)	By 6 mon (*N* = 5,292)	By 12 mon (*N* = 4,052)	By 24 mon (*N* = 2,271)	By 6 mon (*N* = 3,562)	By 12 mon (*N* = 2,718)	By 24 mon (*N* = 1,512)	By 6 mon (*N* = 3,474)	By 12 mon (*N* = 2,647)	By 24 mon (*N* = 1,466)	By 6 mon (*N* = 88)	By 12 mon (*N* = 71)	By 24 mon (*N* = 46)
Time (days) to initiation of first OP medication^1^ (mean days; *SD*)	69.0 (53.0)	120.7 (99.9)	214.3 (198.5)												
≤ 30 days	31.4%	22.4%	17.7%												
> 30–60 days	18.1%	13.7%	9.7%												
> 60–180 days	50.6%	37.1%	28.9%												
> 180–360 days		26.8%	21.4%												
> 360–720 days			22.3%												
Discontinuation	1.1%	2.6%	5.3%	3.8%	9.0%	17.7%	7.2%	15.9%	27.0%	7.3%	16.1%	27.4%	4.5%	9.9%	13.0%
Reinitiation	0.2%	1.3%	3.8%	0.8%	4.7%	12.7%	1.5%	9.0%	21.2%	1.6%	9.1%	21.4%	0.0%	5.6%	13.0%
Post-index OP medication use	11.2%	14.3%	17.6%	40.0%	48.5%	55.0%	68.9%	76.7%	80.8%	68.9%	76.7%	80.8%	70.5%	78.9%	80.4%
Anabolic agents	0.7%	1.0%	1.3%	2.7%	3.3%	4.4%	3.1%	3.6%	4.0%	1.8%	2.2%	2.3%	54.5%	56.3%	58.7%
Teriparatide	0.6%	0.8%	1.1%	2.2%	2.9%	4.0%	2.6%	3.1%	3.7%	1.4%	1.8%	2.0%	50.0%	53.5%	58.7%
Abaloparatide	0.1%	0.2%	0.2%	0.4%	0.4%	0.4%	0.4%	0.4%	0.3%	0.3%	0.4%	0.3%	4.5%	2.8%	0.0%
Evenity	0.0%	0.0%	0.0%	0.0%	0.0%	0.0%	0.0%	0.0%	0.0%	0.0%	0.0%	0.0%	0.0%	0.0%	0.0%
Anti-resorptive agents	10.5%	13.3%	16.3%	37.3%	45.2%	50.6%	65.8%	73.2%	76.8%	67.1%	74.5%	78.5%	15.9%	22.5%	21.7%
Oral BP	6.8%	8.4%	10.2%	21.7%	24.3%	26.3%	43.7%	46.2%	46.6%	44.6%	47.3%	48.0%	4.5%	5.6%	2.2%
IV BP	0.5%	0.9%	1.2%	2.6%	4.4%	5.3%	2.1%	3.6%	4.4%	2.2%	3.7%	4.6%	1.1%	2.8%	0.0%
Calcitonin	1.3%	1.4%	1.6%	2.6%	2.9%	3.2%	3.8%	4.0%	4.2%	3.9%	4.1%	4.4%	0.0%	0.0%	0.0%
Denosumab	1.3%	1.9%	2.4%	8.5%	11.2%	12.5%	10.1%	12.5%	13.8%	10.1%	12.5%	13.6%	9.1%	12.7%	17.4%
SERMs	0.6%	0.7%	0.9%	2.0%	2.4%	3.3%	6.1%	6.9%	7.7%	6.2%	7.0%	7.9%	1.1%	1.4%	2.2%
Occurrence of a subsequent nontraumatic fracture	7.4%	9.9%	13.2%	10.8%	15.9%	22.7%	10.7%	15.3%	21.3%	10.7%	15.1%	20.9%	11.4%	25.4%	34.8%
Hip	0.7%	1.0%	1.5%	1.0%	1.7%	2.6%	0.7%	1.3%	2.3%	0.7%	1.3%	2.3%	0.0%	1.4%	2.2%
Vetebral	3.6%	5.1%	6.6%	6.0%	9.6%	13.3%	5.9%	9.2%	12.3%	5.8%	8.9%	11.8%	10.2%	21.1%	28.3%
NHNV	3.1%	3.9%	5.1%	3.8%	4.7%	6.9%	4.2%	4.8%	6.7%	4.2%	4.9%	6.8%	1.1%	2.8%	4.3%

^1^Time (days) to initiation of first OP medication was reported among OP treatment naïve patients

### Occurrence of new subsequent fracture

Overall, 7.4%, 9.9%, and 13.2% of the population had a new fracture at months 6, 12, and 24 following the index fracture, respectively (Table 4). In patients with an index vertebral fracture, occurrence of a subsequent fracture within months 6, 12, and 24 was 12.5%, 17.7%, and 23.5%, respectively (Table 5).

**Table 5:** Treatment pattern and occurrence of subsequent new fracture by 6, 12, and 24 months following index nontraumatic fracture by type of index fracture

	Subgroup of patients with index hip fracture			Subgroup of patients with index vetebral fracture			Subgroup of patients with index NHNV fracture		
Post index treatment measures	By 6 months (*N* = 5,935)	By 12 months (*N* = 4,490)	By 24 months (*N* = 2,413)	By 6 months (*N* = 14,970)	By 12 months (*N* = 11,243)	By 24 months (*N* = 5,953)	By 6 months (*N* = 28,034)	By 12 months (*N* = 21,522)	By 24 months (*N* = 11,841)
Discontinuation	1.5%	3.8%	7.5%	1.9%	4.3%	8.6%	0.6%	1.4%	3.2%
Reinitiation	0.3%	2.0%	5.6%	0.5%	2.4%	6.3%	0.1%	0.7%	2.2%
Post-index OP medication use	14.2%	19.5%	24.2%	18.7%	22.8%	27.2%	6.5%	8.8%	11.4%
Anabolic agents	1.0%	1.6%	2.1%	1.4%	1.8%	2.3%	0.3%	0.4%	0.6%
Teriparatide	0.8%	1.2%	1.9%	1.2%	1.5%	2.1%	0.2%	0.3%	0.5%
Abaloparatide	0.2%	0.3%	0.2%	0.3%	0.3%	0.3%	0.1%	0.1%	0.1%
Evenity	0.0%	0.0%	0.0%	0.0%	0.0%	0.0%	0.0%	0.0%	0.0%
Anti-resorptive agents	13.3%	18.0%	22.1%	17.2%	21.0%	24.9%	6.3%	8.3%	10.8%
Oral BP	9.8%	12.5%	15.1%	9.8%	11.6%	13.6%	4.6%	5.9%	7.6%
IV BP	0.7%	1.3%	1.8%	0.9%	1.3%	1.7%	0.3%	0.6%	0.9%
Calcitonin	0.6%	0.7%	0.9%	3.6%	3.9%	4.4%	0.2%	0.2%	0.3%
Denosumab	1.6%	2.4%	3.1%	2.3%	3.3%	4.1%	0.7%	1.1%	1.4%
SERMs	0.7%	0.9%	1.2%	0.7%	0.8%	1.1%	0.5%	0.5%	0.7%
Occurrence of a subsequent nontraumatic fracture	10.0%	12.8%	17.4%	12.5%	17.7%	23.5%	4.0%	5.3%	7.1%
Hip	1.3%	2.5%	4.1%	0.5%	0.8%	1.4%	0.7%	0.8%	1.0%
Vetebral	1.1%	1.8%	3.1%	10.0%	14.3%	18.3%	0.6%	0.9%	1.4%
NHNV	7.5%	8.4%	10.2%	2.0%	2.7%	3.8%	2.7%	3.6%	4.8%

## Discussion

This study, to our knowledge, is the first to assess relevant OP-related post fracture management patterns among a nationally representative US sample of commercially insured women. Our findings show that the rates for diagnosis and treatment of OP remained low in commercially insured women with nontraumatic fractures, although an increase was noted in OP diagnosis, treatment and DXA scan following the index nontraumatic fracture remained low, which is consistent with previous finding among commercially insured women [22, 23]. Although the post index OP medication utilization rate among the subgroup of patients with pre-index OP treatment or OP diagnosis is higher, it is still suboptimal. In a previous study, it was also found that discontinuation of OP medications is high in clinical practice due to non-compliance and adverse effects, especially gastrointestinal adverse events [24].

Among patients with OP medication use, a majority of the patients received antiresorptive agents, while only a very small proportion of the patients received anabolic agents. Although AACE guidelines recommended anabolic treatments for very high-risk patients or patients with prior fracture, the use of anabolic treatment is still low in real world setting [12].

Overall, the diagnostic and treatment trend in this study were not in agreement with the current treatment guidelines [12], which strongly recommend that postmenopausal women aged 50 years and older with prior fracture should be treated with pharmacotherapy. This observation is similar to findings from earlier studies on OP management, including studies which showed that only 16% of women aged ≥ 55 and 30% of US Medicare beneficiaries (age > 65) received treatment following a fracture episode [25–27]. There are many factors that may contribute to underdiagnosis and undertreatment of OP in the USA. One potential reason is that patients and physicians do not recognize that osteoporosis and the underlying fragility of bone require screening and treatment. In addition, the OP treatment rate may be lower in working aged commercially insured women compared to Medicare patients due to the misperception that working-aged women are not affected by OP. On the other hand, limited coordination in post-fracture care programs or a lack of coordinated secondary fracture prevention programs may also play a role [28].

The rate of subsequent fracture was 7.4%, 9.9%, and 13.2% within 6, 12, and 24 months following the index fracture. A similar rate of subsequent fracture was observed in previous study of US Medicare and commercially insured beneficiaries 50 and older, which reported 11.6% of the commercially insured beneficiaries experienced a subsequent fracture within 1 year following the index fracture [6]. In this analysis, it was also noted that among those with index vertebral fracture, the subsequent fracture rate is higher than all other types of fracture. Subsequent fracture was reported to be associated with higher medical costs [29], therefore appropriate treatment for OP is needed to further prevent subsequent fracture, reduce the economic burden and individual human suffering.

For the patients with index hip fracture, the orthopedic surgery rate was 90.0% within 6 months on or after the index-fracture, admission rate to a SNF was 96.7%, DME use was 38.1%, and all cause readmission rate within 30 days post discharge of the index fracture hospitalization was 15.1%, which indicates that hip fracture patients require more intensive post fracture care. The length of stay in the SNF and the total number of occupational and physical therapy minutes may be further investigated in future research.

### Limitations

This study has some limitations. Misclassification of patients due to miscoding or misdiagnosis may exist in claims data since these data are mainly for billing and reimbursement purposes, some the medical conditions and outcomes may be documented incorrectly. Secondly, the information of over-the-counter (OTC) treatments such as calcium and vitamin D supplements is lacking in claims data and cannot be captured in this study. Outpatient prescription claims at the health plan level do not necessarily reflect the true utilization of medications. Lastly, underestimation of the subsequent fracture data may exist, given that the requirement of the 90-day gap in the identification of subsequent fractures may not allow inclusion of patients who had a second incident fracture within that time duration. Classification of subsequent vertebral fracture may be overestimated due to the inability in claims data to fully distinguish between the index and subsequent fractures as continuing or separate events. Vertebral fracture is a more chronic condition compared with the more acute fractures such as hip, wrist etc. As the subsequent fracture definition is based on the observation of a subsequent medical claim for a given condition, vertebral fracture was expected to have a higher subsequent fracture (medical claim) rate. While DXA scan orders were captured, DXA values were not available in claims data. Additionally, the number of years from the onset of menopause is unknown, thus an assumption of 50 years old was made for the onset of menopause.

## Conclusion

Findings from this study provide an overview of the post fracture management patterns using real-world data. In this younger (≥ 50 years) patient population (compared with a publicly insured population), OP diagnosis was low which may be due to the very low rate of DXA scans. It was also remarkable that even following a nontraumatic fracture, the rate of osteoporosis treatment was lower than 1 in 8. Nontraumatic fracture, particularly hip fracture, resulted in substantial clinical burden during the post-index period. These data suggest that underdiagnosis and undertreatment exist in a younger commercially insured female population, including women under age 65, even lower than in an older, Medicare fee-for-service population [19]. Therefore, improving osteoporosis management (i.e., screening, diagnosis, and treatment) in younger patients is warranted to reduce fragility fracture among this population.

## Supplementary Information

Below is the link to the electronic supplementary material.

Supplementary file1 (DOCX 33 KB)

## References

[1] Kerr C, Bottomley C, Shingler S, Giangregorio L, de Freitas HM, Patel C, Randall S, Gold DT (2017). The importance of physical function to people with osteoporosis. Osteoporos Int.

[2] Marinho BC, Guerra LP, Drummond JB, Silva BC, Soares MM (2014). The burden of osteoporosis in Brazil. Arq Bras Endocrinol Metabol.

[3] Hansen D, Bazell C, Pelizzari P, Pyenson B (2019) Milliman research report: medicare cost of osteoporotic fractures. The clinical and cost burden of an important consequence of osteoporosis. National Osteoporosis Foundation

[4] Cosman F, de Beur SJ, LeBoff MS, Lewiecki EM, Tanner B, Randall S, Lindsay R, National Osteoporosis F (2014). Clinician’s guide to prevention and treatment of osteoporosis. Osteoporos Int.

[5] Zimmerman S, Chandler JM, Hawkes W, Sloane PD, Hebel JR, Magaziner J, Martin AR, Girman CJ (2002). Effect of fracture on the health care use of nursing home residents. Arch Intern Med.

[6] Weaver J, Sajjan S, Lewiecki EM, Harris ST, Marvos P (2017) Prevalence and cost of subsequent fractures among U.S. patients with an incident fracture. J Manag Care Spec Pharm 23 (4):461–471. 10.18553/jmcp.2017.23.4.461 10.18553/jmcp.2017.23.4.461 PMC10398116 28345441

[7] Johnell O, Kanis J (2005). Epidemiology of osteoporotic fractures. Osteoporos Int.

[8] Pietri M, Lucarini S (2007). The orthopaedic treatment of fragility fractures. Clin Cases Miner Bone Metab.

[9] Budhia S, Mikyas Y, Tang M, Badamgarav E (2012) Osteoporotic fractures: a systematic review of U.S. healthcare costs and resource utilization. Pharmacoeconomics 30 (2):147–170. 10.2165/11596880-000000000-00000. 10.2165/11596880-000000000-00000 22187933

[10] Pisani P, Renna MD, Conversano F, Casciaro E, Di Paola M, Quarta E, Muratore M, Casciaro S (2016). Major osteoporotic fragility fractures: risk factor updates and societal impact. World J Orthop.

[11] Osteoporosis: risk assessment, management and prevention. https://www.nursingtimesnet/clinical-archive/orthopaedics/osteoporosis-risk-assessment-management-and-prevention-01-02-2019/. Accessed Feb 2019

[12] Camacho PM, Petak SM, Binkley N, Diab DL, Eldeiry LS, Farooki A, Harris ST, Hurley DL, Kelly J, Lewiecki EM, Pessah-Pollack R, McClung M, Wimalawansa SJ, Watts NB (2020). American Association of Clinical Endocrinologists/American College of Endocrinology Clinical Practice Guidelines for the diagnosis and treatment of postmenopausal osteoporosis-2020 update. Endocr Pract.

[13] Khan AZ, Rames RD, Miller AN (2018). Clinical management of osteoporotic fractures. Curr Osteoporos Rep.

[14] Iconaru L, Smeys C, Baleanu F, Kinnard V, Moreau M, Cappelle S, Surquin M, Rubinstein M, Rozenberg S, Paesmans M, Karmali R, Bergmann P, Body JJ (2020). Osteoporosis treatment gap in a prospective cohort of volunteer women. Osteoporos Int.

[15] Murphy-Menezes M (2016). The osteoporosis clinical care gap: an opportunity for impact by the clinical pharmacist. Journal of Bone Reports & Recommendations.

[16] Budhia S, Mikyas Y, Tang M, Badamgarav E (2012). Osteoporotic fractures. Pharmacoeconomics.

[17] Becker DJ, Yun H, Kilgore ML (2010). Health services utilization after fractures: evidence from Medicare. J Gerontol A Biol Sci Med Sci.

[18] Kumar A, Rahman M, Trivedi AN, Resnik L, Gozalo P, Mor V (2018) Comparing post-acute rehabilitation use, length of stay, and outcomes experienced by Medicare fee-for-service and Medicare Advantage beneficiaries with hip fracture in the United States: a secondary analysis of administrative data. PLoS Med 15(6):e1002592 10.1371/journal.pmed.1002592 PMC6019094 29944655

[19] Liu J, Gong T, Xu X, Fox KM, Oates M, Gandra SR (2022). Heavy clinical and economic burden of osteoporotic fracture among elderly female Medicare beneficiaries. Osteoporos Int.

[20] Lanteigne A, Sheu YH, Stürmer T, Pate V, Azrael D, Swanson SA, Miller M (2015) Serotonin-norepinephrine reuptake inhibitor and selective serotonin reuptake inhibitor use and risk of fractures: a new-user cohort study among US adults aged 50 years and older CNS drugs 29(3):245–52 10.1007/s40263-015-0231-5 PMC4380622 25708711

[21] Balasubramanian A, Zhang J, Chen L, Wenkert D, Daigle SG, Grauer A, Curtis JR (2019). Risk of subsequent fracture after prior fracture among older women. Osteoporos Int.

[22] Overman RA, Farley JF, Curtis JR, Zhang J, Gourlay ML, Deal CL (2015). DXA utilization between 2006 and 2012 in commercially insured younger postmenopausal women. J Clin Densitom.

[23] Weaver J, Sajjan S, Lewiecki EM, Harris ST (2017). Diagnosis and treatment of osteoporosis before and after fracture: a side-by-side analysis of commercially insured and Medicare advantage osteoporosis patients. J Manag Care Spec Pharm.

[24] Jakob F, Marin F, Martin-Mola E, Torgerson D, Fardellone P, Adami S, ... & Cooper C (2006) Characterization of patients with an inadequate clinical outcome from osteoporosis therapy: the Observational Study of Severe Osteoporosis (OSSO). J Assoc Phys 99(8):531-543 10.1093/qjmed/hcl073 16861718

[25] Boudreau DM, Yu O, Balasubramanian A, Wirtz H, Grauer A, Crittenden DB, Scholes D (2017). A survey of women’s awareness of and reasons for lack of postfracture osteoporotic care. J Am Geriatr Soc.

[26] Yusuf AA, Matlon TJ, Grauer A, Barron R, Chandler D, Peng Y (2016). Utilization of osteoporosis medication after a fragility fracture among elderly Medicare beneficiaries. Arch Osteoporos.

[27] Lewiecki EM, Ortendahl JD, Vanderpuye-Orgle J, Grauer A, Arellano J, Lemay J, Harmon AL, Broder MS, Singer AJ (2019). Healthcare policy changes in osteoporosis can improve outcomes and reduce costs in the United States. JBMR Plus.

[28] Keshishian A, Boytsov N, Burge R, Krohn K, Lombard L, Zhang X, Xie L, Baser O (2017). Examining the treatment gap and risk of subsequent fractures among females with a fragility fracture in the US Medicare population. Osteoporos Int.

[29] Dane Hansen FSA, Pelizzari P, & Bruce Pyenson FSA (2021) Medicare cost of osteoporotic fractures–2021 updated report. The Clinical and Cost Burden of an Important Consequence of Osteoporosis. National Osteoporosis Foundation. Available at https://www.milliman.com/en/insight/-/media/milliman/pdfs/2021-articles/3- 30-21-Medicare-Cost-Osteoporotic-Fractures.ashx

