# Supplementary material for: Medical management patterns in a US commercial claims database following a nontraumatic fracture in postmenopausal women
Source: Arch Osteoporos. 2022 Jul 14;17(1):92. doi: 10.1007/s11657-022-01135-4 (PMC9283183; doi:10.1007/s11657-022-01135-4)
Supplement: Supplementary file 1 — Supplementary file1 (DOCX 33 KB) [file 11657_2022_1135_MOESM1_ESM.docx]

**Medical management patterns in a US commercial claims database following a nontraumatic fracture in postmenopausal women**

Xin Wang,^1^ Xiaoqing Xu,^2^ Mary Oates,^2^ Timothy Hill,^1^ Rolin L. Wade^1^

^1^IQVIA, Plymouth meeting, PA, USA; ^2^Amgen Inc., Thousand Oaks, CA, USA

**Journal:** *Osteoporosis International*

Corresponding author: Xin Wang

Email: [xin.wang0227@gmail.com](mailto:xin.wang0227@gmail.com)

**SUPPLEMENTARY MATERIAL

Supplementary Figure 1. Patient attrition**Flow of patients through sample selection

Women aged ≥50 with a new non-traumatic fracture during the index period (1/1/2015-6/30/2019)

**122,998**

**(100%)**

Patients with continuous enrollment (CE) for both pharmacy and medical benefits for the 1-year pre-index period

**83,111 (67.6%)**

Patient remaining: N=48,939

- Aged 50–64 (n=35400, 72.3%)
- Aged ≥65 (n=13,539, 27.7%)

Patients with CE for both pharmacy and medical benefits for the at least 6- month post-index period

**69,587 (56.6%)**

EXCLUDED PATIENTS (n=20,648)

- Patients with trauma on the same day as the index date
- Patients with non-traumatic fracture during the pre-index period
- Patients with diagnosis of cancers (excluding non-melanoma skin cancer) during the study period
- Patients with diagnosis of Paget's disease of the bone (osteitis deformans), and osteopathies or metabolic bone diseases during the study period
- Patients residing in skilled nursing facility during the pre-index period
- Patients with data quality issue
